# Outcomes of Primary Percutaneous Coronary Intervention in Patients With a Thrombolysis in Myocardial Infarction Score of Five or Higher

**DOI:** 10.7759/cureus.9356

**Published:** 2020-07-23

**Authors:** Kanhiyalal Talreja, Khalil Sheikh, Azizur Rahman, Chander Parkash, Abid Abbas Khan, Faisal Ahmed, Musa Karim

**Affiliations:** 1 Cardiology, Dr. Ruth Pfau Civil Hospital Karachi, Karachi, PAK; 2 Adult Cardiology, National Institute of Cardiovascular Diseases, Karachi, PAK; 3 Adult Cardiology, Civil Hospital Karachi, Pakistan, Karachi, PAK; 4 Statistics, National Institute of Cardiovascular Diseases, Karachi, PAK

**Keywords:** adverse events, primary percutaneous coronary intervention, timi score ≥ 5, acute stemi

## Abstract

Background

Primary percutaneous coronary intervention (PCI) is a treatment of choice for patients with ST-segment elevation myocardial infarction (STEMI). Of the various risk stratification scores that have been introduced, the thrombolysis in myocardial infarction (TIMI) score is among the most used modalities. Patients with a TIMI score of five or higher are classified as high-risk patients with higher rates of adverse events. Therefore, this study aimed to determine the rate of adverse events after primary PCI in patients presenting with STEMI and a TIMI score of five or higher.

Methodology

This descriptive study was conducted at the cardiology department of the Liaquat National Hospital, Karachi, from February 2018 to August 2018. The patients included in this study consisted of a total of 150 men and women who presented to the ED with concerns of chest pain and were diagnosed with STEMI and had a TIMI score of five or higher. Consultant cardiologists performed primary PCI procedures, and any post-procedure adverse events were recorded during the patients’ hospital stays (up to one week), including mortality, heart failure, cardiogenic shock, and ventricular arrhythmias.

Results

The study population was 83.3% male and 16.7% female patients, and the mean age was 54.0 ± 9.4 years. The mean BMI was 27.34 ± 2.76 kg/m^2^. The mean TIMI score was 9.19 ± 2.71, with a TIMI score higher than eight for 52.7% of patients. Death was observed in 18.7% of cases, heart failure in 21.3% of cases, cardiogenic shock in 13.3% of cases, and ventricular arrhythmia in 22.0% of cases.

Conclusion

A TIMI risk score of five or higher can identify patients at high risk not only for mortality, but also for heart failure, cardiogenic shock, and ventricular arrhythmias.

## Introduction

During the twentieth century, there was a decrease in the loss of life and disabilities due to communicable diseases, and non-communicable diseases (NCDs) replaced communicable diseases as the leading cause of death and disability worldwide [1]. As a result, the World Health Organization drafted a global action plan for a 25% reduction in premature deaths due to NCD by 2025, with a focus on prevention, management, and treatment policies for four major NCDs: cardiovascular diseases (CVDs), chronic respiratory diseases, diabetes, and cancer [2]. CVDs currently remain the leading cause of death, and approximately 80% of this burden is contributed by low- and middle-income countries [3,4], such as the South Asian countries. South Asia, which include Pakistan, India, Sri Lanka, Bangladesh, Nepal, Maldives, and Bhutan, comprise one-fourth of the world population are at a markedly increased risk of CVD compared to other western nationalities [5,6]. Although risk factors for CVD are the same throughout the world, lifestyle transitions such as urbanization accompanied by decreased physical activity and increased consumption of tobacco products are contributing to the escalation and progression of CVD in these nations [3]. The biology of atherosclerotic CVD in these nations is not different from that of any other ethnic or racial group. The increased burden of CVD is attributed to the increased prevalence of risk factors such as type II diabetes mellitus, impaired glucose tolerance, metabolic syndrome, and insulin resistance [5].

ST-segment elevation myocardial infarction (STEMI) is a lethal cardiac manifestation that requires acute management and results in high mortality and morbidity. Current management guidelines recommend primary percutaneous coronary intervention (PCI) within 12 hours of symptom onset for patients with STEMI [7]. The main aim of primary PCI is the early restoration of the myocardium. Although innovative management approaches have improved patient outcomes, the identification of high-risk patients, and precise estimation of their prognosis can assist us in further optimization of management strategies. Therefore, effective risk stratification remains an integral part of management strategies [8]. Hence, various risk stratification tools have been introduced for the categorization of patients with STEMI, ranging from some very basic and simple scoring systems to models with a complex interplay of multiple variables [9]. When considering a risk stratification score, there is always a tradeoff between simplicity and accuracy. Consequently, a simple scoring system that can be easily adopted in day-to-day clinical practice with optimized accuracy can be very effective. The thrombolysis in myocardial infarction (TIMI) score is a widely adopted simple system for the risk stratification of STEMI patients, and it has been validated on external cohorts under various clinical settings [9-11].

The beneficence of primary PCI can be different in various subgroups of STEMI patients. Hence, the clinical importance of risk stratification before the intervention is unarguable. In its earlier stages, primary PCI was considered suitable only in low-risk patients. However, recent years have witnessed the benefits of primary PCI in high-risk patients as well. There have been favorable results after primary PCI procedures were performed for patients with risk factors and comorbidities, patients with complex coronary anatomy and severity of disease (such as multivessel involvement, chronic total occlusion, and left main involvement), and patients with hemodynamically unstable clinical status (cardiogenic shock, concomitant valvular disease, and ventricular dysfunction) [12]. In this study, we aimed to determine the rate of adverse events after primary PCI in patients presenting with STEMI with a TIMI score of five or higher.

## Materials and methods

This descriptive study was conducted at the cardiology department of the Liaquat National Hospital, Karachi, during the study period of February 2018 to August 2018. After providing informed consent, a total of 150 patients of both sexes, age 30 to 70 years old, were included in this study. All patients presented to the emergency department with the concerns of chest pain were subsequently diagnosed with STEMI, and underwent primary PCI. The TIMI score was computed as per the scoring system defined by Morrow et al., and all consecutive patients with a TIMI score of five or higher were included in this study [13].

The diagnosis of STEMI was based on the history and electrocardiographic (ECG) assessment of the patient at the time of presentation as per the most recent guidelines [7]. The onset of typical chest pain and associated symptoms in the previous 12 hours that persisted for more than 20 minutes and presentation of ECG changes consistent with the diagnosis of acute STEMI, such as new ST elevation in at least two contiguous leads >2 mm in men or >1 mm in women in leads V2 to V3 and/or >1 mm in other contiguous chest leads or limb leads, were considered for the diagnosis of STEMI. Patients were excluded if they previously received fibrinolytic therapy or were treated with platelet glycoprotein IIb/IIIa inhibitors. Patients were also excluded if they had a previous history of myocardial infarction, thrombolytic therapy, coronary angioplasty, coronary artery bypass grafting surgery, Prinzmetal angina (previous history of cardiac chest pain for less than 20 minutes at rest that occurred in cycles), or cardiogenic shock.

Consultant cardiologists performed the primary PCI procedure. The arterial sheath was passed through the femoral route, the area of occlusion was identified and then was ballooned so that a stent could be inserted. All the patients were kept under observation during their hospital stay (up to one week), and post-procedure adverse events were recorded, which included mortality, heart failure, cardiogenic shock, and ventricular arrhythmias. All the data were recorded on a predefined pro forma document.

The collected data were analyzed with IBM Statistical Package for the Social Sciences (SPSS) Statistics for Windows, Version 21.0 (IBM Corp., Armonk, NY). The mean ± standard deviation and frequency (%) were computed to express the quantitative and qualitative data, respectively. Patients were further divided into two groups based on TIMI score, consisting of a moderate-risk group (patients with a TIMI score between five and eight) and high-risk group (patients with a TIMI score greater than eight). The demographic characteristics and outcome variables were compared between the two groups by applying the chi-square test, and odds ratio (OR), and their 95% confidence interval was computed. Association between post-procedure adverse outcomes and confounding variables, such as sex, age, smoking, hypertension, and diabetes mellitus, was assessed by applying the chi-square test. A P-value ≤0.05 was considered as significant.

## Results

Of 150 patients, 83.3% were male, and 16.7% were female, and the mean age was 54.0 ± 9.4 years. The mean height was 166.3 ± 5.6 cm, the mean weight was 75.6 ± 8.4 kg, and the mean BMI was 27.3 ± 2.7 kg/m^2^. Of the total number of patients, 49.3% were smokers, 66.0% were hypertensive, and 62.7% were diabetic. The mean TIMI score was 9.19 ± 2.71, and it was observed that 47.3% of the cases were at moderate risk, and 52.7% of cases were at high risk. Regarding patient outcomes, death was observed in 18.7% of cases, heart failure in 21.3%, cardiogenic shock in 13.3%, and ventricular arrhythmia in 22.0%. The baseline characteristics, risk factors, and outcomes of primary PCI are presented in Table 1.

**Table 1:** Baseline characteristics, risk factors, and outcomes of primary percutaneous coronary intervention Abbreviations: BMI, body mass index; TIMI, thrombolysis in myocardial infarction

Characteristics	Total
Total	150
Sex	
Male	83.3% (125)
Female	16.7% (25)
Age (years)	54 ± 9.4
≤ 54 years	50.7% (76)
> 54 years	49.3% (74)
BMI (kg/m^2^)	27.34 ± 2.76
< 25 kg/m^2^	16% (24)
25.0-29.9 kg/m^2^	76.7% (115)
> 29.9 kg/m^2^	7.3% (11)
Risk factors	
Smoking	49.3% (74)
Hypertension	66% (99)
Diabetes mellitus	62.7% (94)
TIMI risk score	9.19 ± 2.71
Moderate (5-8)	47.3% (71)
High (≥9)	52.7% (79)
Outcomes	
Mortality	18.7% (28)
Heart failure	21.3% (32)
Cardiogenic shock	13.3% (20)
Ventricular arrhythmia	22% (33)

Table 2 presents the frequency of adverse outcomes according to TIMI risk groups. The results indicate that there was a significant association of ventricular arrhythmia with TIMI risk groups (P = 0.000). Death, heart failure, and cardiogenic shock were insignificantly associated with TIMI risk groups.

**Table 2:** Adverse outcomes of primary percutaneous coronary intervention stratified by thrombolysis in myocardial infarction risk score Abbreviations: BMI, body mass index; TIMI, thrombolysis in myocardial infarction

Outcomes	TIMI Risk Score		P-value
	Moderate Risk	High Risk	
	(5 ≤ TIMI ≥ 8)	(TIMI ≥9)	
Total (N)	71	79	-
Mortality			
Yes	15.5% (11)	21.5% (17)	0.344
No	84.5% (60)	78.5% (62)	
Heart failure			
Yes	19.7% (14)	22.8% (18)	0.647
No	80.3% (57)	77.2% (61)	
Cardiogenic shock			
Yes	14.1% (10)	12.7% (10)	0.798
No	85.9% (61)	87.3% (69)	
Ventricular arrhythmia			
Yes	36.6% (26)	8.9% (7)	<0.001
No	63.4% (45)	91.1% (72)	

The OR for high risk groups was computed as 1.50 (0.65 to 3.46) for death, 1.20 (0.55 to 2.64) for heart failure, 0.88 (0.35 to 2.27) for cardiogenic shock, and 0.17 (0.07 to 0.42) for ventricular arrhythmia. Adverse outcomes of primary PCI by patient characteristics are presented in Table 3.

**Table 3:** Adverse outcomes of primary PCI stratified by patient characteristics Abbreviations: BMI, body mass index; PCI, percutaneous coronary intervention

	Mortality	Heart Failure	Cardiogenic Shock	Ventricular Arrhythmia
Gender				
Male	25 (20.0)	24 (19.2)	15 (12.0)	33 (26.4)
Female	3 (12.0)	8 (32.0)	5 (20.0)	0 (0.0)
P-value	0.415	0.154	0.332	0.004
Age				
≤ 54 years	15 (19.7)	17 (22.4)	8 (10.5)	21 (27.6)
> 54 years	13 (17.6)	15 (20.3)	12 (16.2)	12 (16.2)
P-value	0.733	0.754	0.305	0.092
BMI				
< 25 kg/m^2^	2 (8.3)	5 (20.8)	5 (20.8)	2 (8.3)
25.0- 29.9 kg/m^2^	23 (20.0)	26 (22.6)	12 (10.4)	29 (25.2)
> 29.9 kg/m^2^	3 (27.3)	1 (9.1)	3 (27.3)	2 (18.2)
P-value	0.338	0.724	0.100	0.223
Smoking				
Yes	13 (17.6)	14 (18.9)	7 (9.5)	20 (27.0)
No	15 (19.7)	18 (23.7)	13 (17.1)	13 (17.1)
P-value	0.733	<0.001	0.168	0.142
Hypertension				
Yes	18 (18.2)	23 (23.2)	13 (13.1)	20 (20.2)
No	10 (19.6)	9 (17.6)	7 (13.7)	13 (25.5)
P-value	>0.99	0.429	0.919	0.459
Diabetes mellitus				
Yes	17 (18.1)	20 (21.3)	9 (9.6)	25 (26.6)
No	11 (19.6)	12 (21.4)	11 (19.6)	8 (14.3)
P-value	0.813	0.154	0.079	0.078

## Discussion

The outcomes of primary PCI in patients with STEMI were highly heterogeneous. Among various other factors, patient-specific elements such as risk profile, hemodynamic status at presentation, and disease complexity played a significant role in determining the outcomes of patients. Thus, precise stratification and identification of high-risk individuals are essential for clinical and therapeutic decision making. An ideal risk stratification modality should have not only high predictive accuracy but also should have easy and fast adaptability in day-to-day clinical practice. The TIMI risk score for STEMI is one such simple yet reasonably accurate risk score that can be easily calculated from the data obtained for parameters at the time of presentation and can identify patients with high risk [13]. The TIMI score was previously developed and validated in a randomized controlled trial of patients treated with fibrinolysis. However, a primary PCI registry and observational studies have reported the satisfactory predictive utility of the TIMI score in patients treated with primary PCI [8].

Patients with a TIMI score of five or higher are considered to be high-risk patients. Hence, in this study, we assessed post-primary PCI adverse events in patients with a TIMI score of five or higher. There is an increased incidence of adverse events in patients with a TIMI score of five or higher as compared to patients with a TIMI score of less than five. A study conducted by González-Pacheco et al. compared the adverse events between the two groups and reported significantly higher rates of mortality, heart failure, cardiogenic shock, and ventricular arrhythmias with rates of 14.8% vs. 2.1%, 15.3% vs. 4.1%, 10.9% vs. 1.5%, and 14.8% vs. 5.9%, respectively [14].

In our study of 150 patients, 47.3% of the cases were at moderate risk (5 ≤ TIMI ≥ 8), and 52.7% cases were stratified as high risk (TIMI ≥9). In our study, the rates of adverse events such as death, heart failure, cardiogenic shock (CS), and ventricular arrhythmia were 18.7%, 21.3%, 13.3%, and 22.0%, respectively. A study conducted by Iltaf et al. reported a TIMI risk score of nine or higher in 31.84% of the patients undergoing primary PCI [15]. The study further reported higher rates of adverse events and complications in patients with TIMI ≥ 9, such as death, stroke, pulmonary edema, and CS at 72.7%, 72.72%, 60.6%, and 72.64%, respectively. The rate of adverse events in this cohort was relatively much higher than the outcomes of primary PCI that were previously reported in large registry-based studies from our population. The reported in-hospital rate of mortality and heart failure ranged from 2.2% to 3.04% and 0.7% to 0.9%, respectively, with cardiogenic shock up to 1.3% [16-18].

Furnaz et al. conducted a study in our population to assess the performance of the TIMI score in elderly female patients and reported a strong linear relationship between the TIMI score and mortality rate [10]. It was observed that there was a mortality rate of 5.6% with a TIMI score of less than five and a mortality rate of 54.4% with a TIMI score of eight or higher. The study authors also performed a receiver operating characteristic curve analysis and reported an area under the curve value of 0.709 (0.591-0.827) as a predictive value of the TIMI score for in-hospital mortality after primary PCI.

The TIMI score is advantageous because it is a simple and easy to use scoring system. However, its clinical utility and effectiveness as a prognostic marker are questionable, especially in the setting of an acute coronary syndrome. A meta-analysis of 10 prospective cohort studies reported a strong linear relationship between the incidence of adverse events and TIMI score. However, a 30-day cardiac event rate of 1.8% was also observed among patients with a TIMI score of zero. Hence, it has been recommended to use it as an adjunct to clinical acumen, and it should not be the sole determinant of patient disposition [19].

Similarly, various studies have compared the TIMI score with other multiple risk stratification models such as the Global Registry of Acute Cardiac Events (GRACE), HEART score, Controlled Abciximab and Device Investigation to Decrease Late Angioplasty Complications (CADILLAC), Zwolle (ZRS), and Primary Angioplasty in Myocardial Infarction (PAMI) [20-23]. The HEART score was reported to outperform the GRACE and TIMI score in discriminating Major Adverse Cardiac Events (MACE) within six weeks in patients presenting with chest pain [21]. The prognostic capacity of the GRACE score was reported to be superior to that of the TIMI score in discriminating in-hospital events in patients with acute coronary syndrome [22]. Similarly, the GRACE score was found to be more appropriate than the TIMI, CADILLAC, PAMI, and Zwolle scores for long-term follow-up [20].

Our study was limited by a lack of comparative control groups (low-risk group) and a lack of comparison with other well-established risk stratification models such as HEART, GRACE, CADILLAC, PAMI, and Zwolle scores. Another important limitation of this study was lack of post-discharge follow-up, in this study we followed patients till their discharge from hospital, while, TIMI score predicts post-procedure 30 days outcome in STEMI patients. Hence, additional prospective and comparative studies are required to identify the optimal risk stratification model for this population.

## Conclusions

The TIMI score is an important risk stratification model. In the current study, STEMI patients with a TIMI score of five or higher exhibited higher rates of post-primary PCI adverse outcomes such as mortality, heart failure, cardiogenic shock, and ventricular arrhythmias. The TIMI score can be used as an adjunct to clinical acumen for effective clinical decision-making.
